# Legal needs of patients attending an urban family practice in Hamilton, Ontario, Canada: an observational study of a legal health clinic

**DOI:** 10.1186/s12875-020-01339-y

**Published:** 2020-12-12

**Authors:** Gina Agarwal, Melissa Pirrie, Dan Edwards, Bethany Delleman, Sharon Crowe, Hugh Tye, Jayne Mallin

**Affiliations:** 1 Department of Family Medicine, McMaster University, Hamilton, Canada; 2 Department of Health Research Methods, Evidence, and Impact, Hamilton, Canada; 3 McMaster Family Practice, Hamilton Health Sciences, Hamilton, Canada; 4 Hamilton Community Legal Clinic, Hamilton, Canada; 5 Legal Aid Ontario, Hamilton, Canada

**Keywords:** Primary care, Medical-legal partnership, Legal needs, Social determinants of health, System navigation, Poverty

## Abstract

**Background:**

Individuals living in poverty often visit their primary care physician for health problems resulting from unmet legal needs. Providing legal services for those in need may therefore improve health outcomes. Poverty is a social determinant of health. Impoverished areas tend to have poor health outcomes, with higher rates of mental illness, chronic disease, and comorbidity. This study reports on a medical-legal collaboration delivered in a healthcare setting between health professionals and lawyers as a novel way to approach the inaccessibility of legal services for those in need.

**Methods:**

In this observational study**,** patients aged 18 or older were either approached or referred to complete a screening tool to identify areas of concern. Patients deemed to have a legal problem were offered an appointment at the Legal Health Clinic, where lawyers provided legal advice, referrals, and services for patients of the physicians. Fisher’s exact test was used to compare populations. Binary logistic regression was used to determine the factors predicting booking an appointment with the clinic.

**Results:**

Eighty-four percent (*n* = 648) of the 770 patients screened had unmet legal needs and could benefit from the intervention, with an average of 3.44 (SD = 3.42) legal needs per patient screened. Patients with legal needs had significantly higher odds of attending the Legal Health Clinic if they were an ethnicity that was not white (OR = 2.48; 95% CI 1.14–5.39), did not have Canadian citizenship (OR = 4.40; 95% CI 1.48–13.07), had housing insecurity (OR = 3.33; 95% CI 1.53–7.24), and had difficulty performing their usual activities (OR = 2.83; 95% CI 1.08–7.43). As a result of the clinic consultations, 58.0% (*n* = 40) were referred to either Legal Aid Ontario or Hamilton Community Legal Clinic, 21.74% (*n* = 15) were referred to a private lawyer; one case was taken on by the clinic lawyer.

**Conclusion:**

The Legal Health Clinic was found to fulfill unmet legal needs which were abundant in this urban family practice. This has important implications for the future health of patients and clinical practice. Utilizing a Legal Health Clinic could translate into improved health outcomes for patients by helping overcome barriers in accessing legal services and addressing social causes of adverse health outcomes.

## Background

Patients living in poverty often consult with their family physicians for assistance with health problems that are the result of unidentified and unmet legal needs [1]. Data from the USA and Canada suggests that people of low income may have two or three unmet legal needs [2–4]. Though family physicians may know about this association between poverty, legal issues, and health, they are unable to help their patients with their legal issues due to a lack of legal knowledge, time and resources [5]. The inaccessibility of legal services to low income individuals could be considered a social determinant of health [6, 7]. In contrast, providing access to justice for those in need may improve poverty and therefore health outcomes [4, 7]. Possible legal mechanisms for reducing poverty include: appeasing debtors, accessing available benefit programs, preventing eviction from housing, addressing unsafe housing conditions, seeking court action to gain spousal support, and settling other court actions [2, 6]. Breaking the cycle of poverty requires dedicated professionals working together as a team to give the patient/client opportunities and support [7]. Therefore, the healthcare setting is an ideal situation for this type of inter-professional collaboration to occur [7].

In 2016, 13% or 4.5 million Canadians were living in poverty [8] based on the Low Income Measure (LIM) [9]. Poverty is widely recognized as a social determinant of health [10, 11]. The World Health Organization describes social determinants of health as “the conditions in which people are born, grow, live, work and age,” factors that are heavily influenced by wealth distribution, power and resources [11, 12]. Due to this direct link between poverty and health, it is not surprising that poverty is a serious problem identified in primary care, especially in low socioeconomic status (SES) communities [5]. For example, insufficient income can result in lack of access to medicines and untreated or sub-optimally treated chronic illness [13, 14]. Poor diet and stressful environments can lead to poor lifestyle choices and, as a result, mental illness, chronic disease and multiple co-morbidities that affect overall quality of life [13, 14].

Medical-legal collaborations delivered in a healthcare setting between health professionals and lawyers can present a novel way to approach these types of problems, by addressing the legal needs that influence poverty [15, 16]. These partnerships can reduce stress and improve the wellbeing of clients through increased access to resources and better awareness of social services as shown in limited evidence from the USA [1, 17]. Medical-legal partnerships of differing varieties have been adopted in more than three hundred health care delivery situations in the USA, [18] though first developed at Boston Medical Center in 1993 [7]. In 2006 a National Centre for Medical-Legal Partnership was created; legal-aid agencies, pro-bono lawyers, and law schools partner with hospitals and health clinics, offering assistance with patients’ unmet legal needs in health care settings [7]. However, medical-legal partnerships are a new concept in Canada and while some collaborations already exist in pediatric hospitals, [2] they have not been rigorously evaluated in a primary care setting. Thus, it is necessary to develop, implement and evaluate such partnerships to inform future medical-legal partnerships in Canada.

With 15% of residents below the LIM definition of poverty [19] and a high rate of emergency shelter use, [20] Hamilton, Ontario is an area of low SES. Hamiltonians’ health is affected by poverty such that there is a significant disparity in life expectancy, with individuals in poor areas expected to live 21 years fewer than those living in wealthier areas [21]. Chronic disease rates are slightly higher than those in Canada as a whole, and lifestyle behaviors are slightly worse [22]. Hamilton is therefore an optimal urban location for the implementation and evaluation of a medical-legal partnership.

This paper reports the development of a novel legal assessment clinic embedded within primary care and explores the following research questions:

1. What are the types of legal needs identified when screening in a primary care setting?
2. What are the characteristics of the population with legal needs in primary care and are they significantly different from those without legal needs in the same setting?
3. What characteristics are associated with individuals choosing to access the legal health clinic?

## Methods

### Research design

A Legal Health Clinic (LHC) in an urban primary care setting was developed and implemented. A cross-sectional observational design was used to evaluate the amount of legal need in primary care, the characteristics of those with legal needs and of LHC attendees. Ethical clearance was granted by the Hamilton Integrated Research Ethics Board.

### Participants and setting

Patients aged 18 years or over were either approached in the family practice waiting room by a research assistant to complete a screening tool (Legal Health Check-Up survey) [1] or were referred to the LHC by their primary care doctor or other healthcare staff within the clinic, and then completed the screening tool. For patients not screened in the waiting room, referral criteria utilised by the family doctor or other healthcare staff were that the patient needed to have a potential legal need such as an upcoming eviction, that had been discussed with the healthcare provider and was concerning for their health outcomes. These patients would then need to complete the screening tool. There were no exclusion criteria, however participants needed to bring their own translators if they did not speak English.

The study was set in a primary care medical clinic within a family health team (FHT) with approximately 13,000 patients, in Hamilton, Ontario.

### Development of the legal health clinic

This study involved providing legal aid services, in the form of a weekly clinic for patients of the FHT’s physicians. The legal clinic was created through a three-way partnership: between the FHT, Hamilton Community Legal Clinic (HCLC) and Legal Aid Ontario (LAO). A lawyer from each legal partner was onsite in the FHT clinical space, every week, on an alternating basis. The lawyers provided legal advice on multiple domains of law. The HCLC lawyer had expertise with housing, employment, social assistance, and human rights issues while the LAO lawyer had expertise on criminal, family, refugee, and estate law. As a result, the McMaster Family Practice LHC became available as a free service to patients of the FHT during this study, with the intention to continue afterwards if it was deemed feasible.

### Data collection

The Legal Health Check-Up survey [23] was used as a screening tool to identify areas of possible concern and to initiate a conversation with participants about legal problem areas. It contained questions about stability of income and housing, benefit status, existence of a will, pending legal worries and discrimination or human rights issues, to name a few. Legal needs were those housing, financial, employment, social assistance, immigration, benefit needs, and other concerns that involved legal processes that either the patient did not have access to or did not understand. In collaboration with LAO, HCLC, and the FHT’s system navigator, an algorithm was developed to determine if the concern identified was a legal issue or if it was a non-legal issue that could be better addressed by the system navigator. Participants identified to have a legal issue were offered an appointment with the LHC, but could also elect to handle the problem on their own, with or without the help of the system navigator. Participants wanting to pursue legal help were matched to a lawyer in the LHC with experience in the appropriate legal domain (e.g. housing).

In conjunction with the Legal Health Check-Up survey, participants also completed a study survey asking their demographics (age, gender, marital status, educational status, income level, citizenship status and ethnicity), quality of life (EQ-5D-3L), [24] self-reported health status, [25] benefit status and poverty indicators not already captured in the Legal Health Check-Up survey. The three added poverty indicator questions have been validated for use in healthcare settings [26, 27] and measured income security (“Do you have difficulty making ends meet at the end of the month?”), [26] housing security (“Do you ever worry about losing your place to live?“), [26] and food security (“In the past month, was there any day when you or anyone in your family went hungry because you did not have enough money for food?”) [26, 27].

The LIM 50 is a common poverty threshold used internationally [9]. The indicator determines if the individual’s household income is below the median household income in their country, adjusted for their household size [9]. Since household size was not included in the baseline surveying, LIM 50 could not be calculated for the full sample. However, individuals who opted to complete the follow-up survey (open to all baseline participants) provided household size, which allowed LIM 50 to be calculated for those individuals. Aside from household size, data collected through follow-up surveying is not presented in this paper.

### Intervention: legal health clinic

At the LHC appointment, participants were scheduled for a 30-min consultation with one of the two lawyers. There were several possible outcomes. Some participants would be provided with resources or educated about an area of law and that would be sufficient to either solve or help with their legal problem. Participants who needed more assistance would be referred to one of four options: 1) LAO, if their income was low enough to qualify for Legal Aid services [28]; 2) HCLC, if their income was low enough to qualify for HCLC services; 3) the LHC lawyer, if they opted to take on the case; or, 4) a private lawyer, for those financially ineligible for LAO or HCLC services due to higher income. It was then up to the participant to pursue the help recommended to them by the LHC. Participants were able to return to the LHC if they wanted more advice or had a new legal problem.

Information on the Legal Health Check-up survey was uploaded to the participants’ medical charts automatically. If the participant provided written consent, information from their legal appointment was added to their electronic medical record, visible to their family physician. The study research assistant scanned the paper documentation and uploaded it to the participant’s electronic medical record. Lawyers were not given access to the participant’s medical record. If the participant agreed, the legal team communicated with the medical team to arrange necessary items such as physical examinations for the Workers Insurance Safety Board. The lawyers would also recommend that the participant visit other services within the FHT, such as the system navigator, where appropriate.

### Data analysis

Measures were assessed using descriptive analysis, including the number of screening surveys completed, number of LHC appointments and subsequent referrals, and types of legal issues identified. Rates and frequencies of demographic characteristics of those who completed the LHC screening survey, of those with unmet legal needs, of those who made an appointment for the LHC and of those who kept their appointment with the LHC were calculated. Where appropriate chi-squared or Fisher’s Exact tests were used to compare populations. Binary logistic regression was completed to determine the factors predicting booking an appointment with the LHC. All statistical tests were performed on SPSS versions 20 and 24.

## Results

In the first 6 months of the program, between April 25, 2016 and October 24, 2016, 770 patients from the FHT consented to participate in the study and completed the Legal Health Check-Up survey. Almost all participants were recruited while waiting as patients in the clinic waiting room (98.4%, *n* = 758) and the remaining participants were patients referred directly to the LHC by another source (1.6%, *n* = 12). Of the 12 participants referred directly to the LHC, five were referred by their physician within the FHT, five by the FHT system navigator, and two by other patients.

### Survey participant demographics

The demographic characteristics of all participants who completed the study survey (*N* = 770), of those who had at least one legal need (*n* = 648, 84.2%), and of those who had no legal need (*n* = 122, 15.8%) are summarized in Table 1. The majority of survey respondents were female (65.6%), had post-secondary education (59.4%), owned their residence (51.0%), were Canadian citizens (92.8%), were white (82.1%), and did not report receiving financial benefits (e.g. ODSP; 55.5%). Respondents were most commonly employed full-time (39.4%) and had a monthly income above $3000 (44.3%). The 18–24 year old age category had the lowest representation (8.4%) and the 35–44 year old age category had the highest representation (22.5%).

**Table 1:** Socio-demographic characteristics of participants with and without legal needs and comparisons between these two groups

	All survey respondents ***N*** = 770	At least one legal need^1^ ***n*** = 648	No legal needs ***n*** = 122	Has legal need versus No legal needs
	n (%)	n (%)	n (%)	*p* value^2^
**Age**				
18–24	64 (8.4)	63 (9.8)	1 (0.8)	*p* < 0.001
25–34	143 (18.7)	132 (20.5)	11 (9.2)	
35–44	172 (22.5)	156 (24.2)	16 (13.3)	
45–54	156 (20.4)	138 (21.4)	18 (15.0)	
55–64	116 (15.2)	86 (13.4)	30 (25.0)	
65 and older	113 (14.8)	69 (10.7)	44 (36.7)	
Total	764	644	120	
**Gender**				
Female	505 (65.6)	433 (66.8)	72 (59.0)	*p* = 0.188
Male	255 (33.1)	207 (31.9)	48 (39.3)	
Transgender	10 (1.3)	8 (1.2)	2 (1.6)	
Total	770	648	122	
**Education**				
University or college graduate	442 (59.4)	353 (56.4)	89 (75.4)	*p* = 0.001
Some college or university	149 (20.0)	132 (21.1)	17 (14.4)	
High School	117 (15.7)	107 (17.1)	10 (8.5)	
Less than high school	36 (4.8)	34 (5.4)	2 (1.7)	
Total	744	626	118	
**Employment**				
Employed, full time	293 (39.4)	244 (38.9)	49 (42.2)	*p* < 0.001
Employed, part time	131 (17.6)	119 (19.0)	12 (10.3)	
Unemployed	93 (12.5)	89 (14.2)	4 (3.4)	
Retired	121 (16.3)	73 (11.6)	48 (41.4)	
Unable to work	105 (14.1)	102 (16.3)	3 (2.6)	
Total	743	627	116	
**Monthly Household Income**				
Less than $650.00	29 (4.0)	28 (4.6)	1 (0.9)	*p* < 0.001
$700.00 to $1800.00	217 (29.9)	210 (34.4)	7 (6.1)	
$1850.00 to $3000.00	158 (21.8)	141 (23.1)	17 (14.9)	
Above $3000.00	321 (44.3)	232 (38.0)	89 (78.1)	
Total	725	611	114	
**Benefits**				
CPP-R	75 (9.7)	46 (7.1)	29 (23.8)	*p* < 0.001
CPP-D / ODSP	101 (13.1)	99 (15.3)	2 (1.6)	
EI / EI Sick Benefits	47 (6.1)	44 (6.8)	3 (2.5)	
Other (e.g. Ontario Works)	120 (15.6)	107 (16.5)	13 (10.7)	
No response	427 (55.5)	352 (54.3)	75 (61.5)	
Total	770	648	122	
**Housing**				
Owns residence	392 (51.0)	288 (44.5)	104 (85.2)	*p* < 0.001
Rents residence	289 (37.6)	277 (42.8)	12 (9.8)	
Lives with friends or family	67 (8.7)	62 (9.6)	5 (4.1)	
Other	21 (2.7)	20 (3.1)	1 (0.8)	
Total	769	647	122	
**Relationship Status**				
Married	321 (42.7)	240 (37.9)	81 (68.1)	*p* < 0.001
Common law or cohabiting	94 (12.5)	87 (13.7)	7 (5.9)	
Single (never married)	178 (23.7)	168 (26.5)	10 (8.4)	
Widowed	33 (4.4)	24 (3.8)	9 (7.6)	
Divorced	73 (9.7)	64 (10.1)	9 (7.6)	
Separated	53 (7.0)	50 (7.9)	3 (2.5)	
Total	752	633	119	
**Citizenship Status**				
Canadian Citizen	691 (92.8)	576 (91.9)	115 (97.5)	*p* = 0.032
Other	54 (7.2)	51 (8.1)	3 (2.5)	
Total	745	627	118	
**Race**				
White/Caucasian	612 (82.1)	498 (79.3)	114 (97.4)	*p* < 0.001
Other	133 (17.9)	130 (20.7)	3 (2.6)	
Total	745	628	117	

Notes: ^1^ Participants with legal needs indicated by the Legal Health Check-Up survey; ^2^ *p*-value from Fisher’s Exact Test; *CPP-R* Canadian Pension Plan, retired, *CPP-D* Canadian Pension Plan, disability, *ODSP* Ontario Disability Support Plan, *EI* Employment Insurance

When comparing those who had a legal need to those who did not, using Fisher’s Exact tests, all demographic variables showed a significant difference (*p* < 0.05), except for gender (*p* = 0.188) (see Table 1). Those who had a legal need were predominantly younger, had attained lower education, had lower monthly income, did not own their residence, were not married, did not have Canadian citizenship, and were not white. Of note, the rate of full-time employment was similar between those with and without a legal need (38.9% versus 42.2%), however those without a legal need had a higher rate of retirees (41.4% versus 11.6%) while those with a legal need had a higher rate of part-time employment, unemployment, and inability to work (49.5% versus 16.3%). Similarly, those who did not have a legal need had a higher rate of receiving Canada Pension Plan (CPP) retirement benefits (23.8% versus 7.1%), while those with a legal need had higher rates of receiving disability, employment, and other benefits (38.6% versus 14.8%).

### Poverty indicators, self-reported health status, and quality of life

The 770 participants who completed the study survey predominantly screened negative for each poverty indicator; 38.1% (*n* = 293) had income insecurity, 21.9% (*n* = 169) could not afford their medication, 12.2% (*n* = 89) could not afford food, and 11% (*n* = 80) had housing insecurity (see Table 2). In the subset of 283 participants who had household size available from a follow-up survey, 52.7% (*n* = 149) had a household income below LIM-50, indicating that their household income fell into the poverty bracket. Self-reported Health Status for the full survey sample was Very Good or Excellent in 40.4% (*n* = 298) of respondents. The EQ-5D-3L tool measuring five domains of quality of life showed that 28.5% (*n* = 208) had mobility issues, 8.0% (*n* = 58) had difficulties with self-care, 34.7% (*n* = 253) had issues performing usual activities, 60.6% (*n* = 443) had issues with pain or discomfort, and 51.9% (*n* = 376) had some or severe anxiety or depression.

**Table 2:** Poverty indicators, self-reported health status, and quality of life in participants with and without legal needs and comparisons between these two groups

	All survey respondents ***N*** = 770	At least one legal need^1^ ***n*** = 648	No legal needs ***n*** = 122	Has legal need versus No legal needs
	n (%)	n (%)	n (%)	*p*-value^2^
**Household Income: Low-Income Measure 50**^**3**^				
Above LIM 50^3^	134 (47.3)	86 (38.1)	48 (84.2)	*p* < 0.001
Below LIM 50^3^	149 (52.7)	140 (61.9)	9 (15.8)	
Total	283	226	57	
**Income Insecurity**				
Yes	293 (38.1)	293 (45.2)	0 (0.0)	Not applicable^4^
No	477 (61.9)	355 (54.8)	122 (100)	
Total	770	648	122	
**Cannot afford medication**				
Yes	169 (21.9)	165 (25.5)	4 (3.3)	*p* < 0.001
No	601 (78.1)	483 (74.5)	118 (96.7)	
Total	770	648	122	
**Food Insecurity**				
Yes	89 (12.2)	88 (14.3)	1 (0.9)	*p* < 0.001
No	641 (87.8)	529 (85.7)	112 (99.1)	
Total	730	617	113	
**Housing Insecurity**				
Yes	80 (11.0)	77 (12.6)	3 (2.7)	*p* = 0.001
No	645 (89.0)	536 (87.4)	109 (97.3)	
Total	725	613	112	
**Self-reported Health Status**				
Excellent/Very good	298 (40.4)	226 (36.4)	72 (62.1)	*p* < 0.001
Good/Fair	379 (51.4)	335 (53.9)	44 (37.9)	
Poor	60 (8.1)	60 (9.7)	0 (0.0)	
Total	737	621	116	
**Quality of Life**				
**Mobility**				
No problem	522 (71.5)	426 (69.4)	96 (82.8)	*p* = 0.003
Some/Severe problems	208 (28.5)	188 (30.6)	20 (17.2)	
Total	730	614	116	
**Self-care**				
No problem	670 (92.0)	557 (90.9)	113 (98.3)	*p* = 0.004
Some/Severe problems	58 (8.0)	56 (9.1)	2 (1.7)	
Total	728	613	115	
**Performing usual activities**				
No difficulty	477 (65.3)	379 (61.7)	98 (84.5)	*p* < 0.001
Some/Severe difficulty	253 (34.7)	235 (38.3)	18 (15.5)	
Total	730	614	106	
**Pain and discomfort**				
None	288 (39.4)	236 (38.4)	52 (44.8)	p = 0.214
Some/Severe	443 (60.6)	379 (61.6)	64 (55.2)	
Total	731	615	116	
**Anxiety and depression**				
None	349 (48.1)	271 (44.4)	78 (68.4)	p < 0.001
Some/Severe	376 (51.9)	340 (55.6)	36 (31.6)	
Total	725	611	114	

Notes: ^1^ Participants with legal needs indicated by the Legal Health Check-Up survey; ^2^ *p*-value from Fisher’s Exact Test; ^3^Low Income Measure 50 (LIM-50) is the threshold for median (50th percentile) household income, adjusted for household size. Household size was asked on a follow-up survey, not baseline, so LIM-50 could only be calculated for 283 participants. ^4^Income insecurity was considered a legal need on the screening survey, hence this poverty indicator had a frequency of 0% for those without a legal need and it was not appropriate to run significance testing for this measure

When comparing those who had a legal need to those who did not, using Fisher’s Exact tests, all poverty, health status, and quality of life variables showed a significant difference (*p* < 0.05), except for pain and discomfort (*p* = 0.214) (see Table 2). Those who had a legal need had a higher rate of inability to afford medication (25.5% versus 3.3%), inability to afford food (14.3% versus 0.9%), and housing insecurity (12.6% versus 2.7%). In the subset of 283 participants who had household size available from a follow-up survey, 61.9% (*n* = 140) of those with a legal need had a household income below LIM-50, compared to 15.8% (*n* = 9) of those without a legal need. In those with a legal need, 9.7% (*n* = 60) had a self-reported health status of ‘poor’ compared to 0% of those without a legal need. Also, the most frequent response for self-reported health status was ‘fair or good’ among those with a legal need (53.9%, *n* = 335), while the most frequent response was ‘very good or excellent’ among those without a legal need (62.1%, *n* = 72). Finally, the EQ-5D-3L tool measuring five domains of quality of life showed those with a legal need more frequently had difficulty with mobility (30.6% versus 17.2%), self-care (9.1% versus 1.7%), performing usual activities (38.3% versus 15.5%), and anxiety/depression (55.6% versus 31.6%).

### Legal needs in primary care

**Fig. 1:**
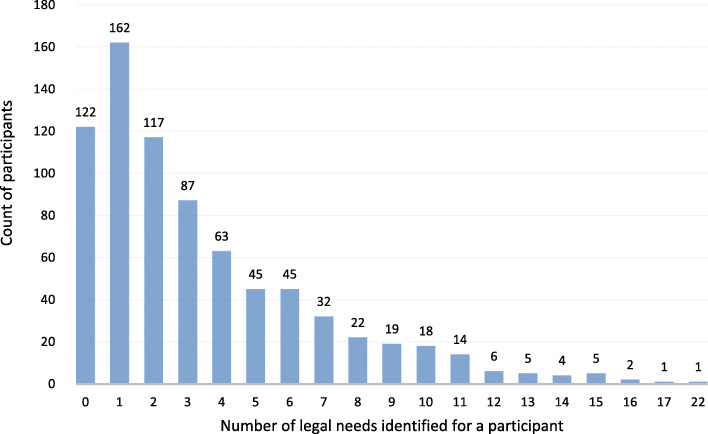
Distribution of legal needs (*n* = 2648) across participants (*n* = 770) as identified through Legal Health Check-up survey screening

Using the Legal Health Check-Up tool, 2648 legal needs were identified through screening and 648 (84.2%) participants had at least one legal need. There was an average of 3.44 (SD = 3.42) legal needs per participant screened and an average of 4.09 (SD = 3.35) legal needs per participant who had at least one legal issue; see Fig. 1 for the distribution of legal needs across participants screened. The types of legal needs identified were family/community (82.9% of respondents, *n* = 537), income (56%, *n* = 363), employment (46.9%, *n* = 304), housing (40.3%, *n* = 261), and health (34.9%, *n* = 226). Details on the subcategories for each of these five types of legal needs and their frequencies can be seen in Table 3. Notably, 75.0% (*n* = 486) of respondents did not have a will, 45.2% (*n* = 293) had trouble making ends meet, 31.2% (*n* = 202) did not have someone to make health decisions if they become unable, 30.1% (*n* = 195) had been hurt at work, 24.8% (*n* = 161) needed help getting or keeping their benefits, and 24.1% (*n* = 156) were in a relationship where someone was trying to control them.

**Table 3:** Types of legal needs identified using the Legal Health Check-up Survey

Indicated at least one legal need (n = 648)	
Type of Legal Need	n (%)
**Income**	**363 (56.0)**^**a**^
‘Trouble making ends meet’	293 (45.2)
Needing help getting or keeping benefits	161 (24.8)
Someone taking their money or possessions without permission	42 (6.5)
Medical review date for ODSP	18 (2.8)
Other/unspecified income issue	92 (14.2)
**Housing**	**261 (40.3)**^**a**^
Problems with home repairs, heat not working, bed bugs, etc.	112 (17.3)
Late with rent this year	85 (13.1)
Problems with neighbours	75 (11.6)
Denied a rental unit due to discrimination	75 (11.6)
Being harassed or discriminated by their landlord	32 (4.9)
Worried rent subsidy will be cancelled	29 (4.5)
Behind with rent	25 (3.9)
Served eviction papers	24 (3.7)
Court order affecting where or with whom they can live	21 (3.2)
Being threatened with eviction	16 (2.5)
Other/unspecified housing issue	45 (6.9)
**Employment**	**304 (46.9)**^**a**^
Has been hurt at work	195 (30.1)
Having trouble finding work due to discrimination	107 (16.5)
Has been harassed by your employer or colleagues	40 (6.2)
Current or past employer owes them money	22 (3.4)
Other/unspecified employment issue	53 (8.2)
**Health**	**226 (34.9)**^**a**^
Does not have someone to make health decisions if they are unable to do so	202 (31.2)
Other/unspecified health issue	46 (7.1)
**Family/Community**	**537 (82.9)**^**a**^
Does not have a will	486 (75.0)
Relationship where someone tries to control them	156 (24.1)
Going through a divorce or separation	63 (9.7)
Problems with child support or access	54 (8.3)
Trouble bringing family members to Canada	29 (4.5)
Trouble attaining citizenship	15 (2.3)
Other/unspecified family or community issue	35 (5.4)

*Note:* ^*a*^ Respondent indicated at least one item from the respective list

The Legal Health Check-Up tool also identified that all 770 participants screened (100%) had at least one issue related to social determinants of health that could be referred to the system navigator (e.g. assistance accessing subsidized daycare). A total of 5371 issues were identified through screening and there was a mean of 6.98 issues (SD = 4.29) per participant screened.

### Legal clinic attendees

Of the 648 participants identified through screening to have a legal need, 94 (14.5%) booked an appointment with the LHC; of the 94 who booked, 69 (73.4%) attended the LHC once and five (5.3%) attended twice. In Table 4, the socio-demographic characteristics of those who booked and those who attended the LHC are provided. Since the objective of this study was not to compare these groups, there were no statistical comparisons made, however the frequencies for each group have been reported for transparency and completeness. Similarly, the poverty indicators, self-reported health status, quality of life, and types of legal needs have been reported for both of these groups in Tables 5 and 6.

**Table 4:** Socio-demographic characteristics of participants who booked and attended Legal Health Clinic appointments

	Booked appointment ***n*** = 94	Attended appointment ***n*** = 69
	n (%)	n (%)
**Age**		
18–24	9 (9.8)	5 (7.4)
25–34	19 (20.7)	12 (17.6)
35–44	24 (26.1)	17 (25.0)
45–54	21 (22.8)	17 (25.0)
55–64	12 (13.0)	10 (14.7)
65 and older	7 (7.6)	7 (10.3)
Total	92	68
**Gender**		
Female	57 (60.6)	41 (59.4)
Male	34 (36.2)	25 (36.2)
Transgender	3 (3.2)	3 (4.3)
Total	94	69
**Education**		
University or college graduate	35 (40.7)	25 (41.0)
Some college or university	24 (27.9)	16 (26.2)
High School	17 (19.8)	14 (23.0)
Less than high school	10 (11.6)	6 (9.8)
Total	86	61
**Employment**		
Employed, full time	13 (14.6)	7 (10.9)
Employed, part time	16 (18.0)	14 (21.9)
Unemployed	21 (23.6)	16 (25.0)
Retired	8 (9.0)	8 (12.5)
Unable to work	31 (34.8)	19 (29.7)
Total	89	64
**Monthly Household Income**		
Less than $650.00	10 (11.1)	6 (9.2)
$700.00 to $1800.00	51 (56.7)	40 (61.5)
$1850.00 to $3000.00	20 (22.2)	13 (20.0)
Above $3000.00	9 (10.0)	6 (9.2)
Total	90	65
**Benefits**		
CPP-R	5 (5.3)	4 (5.8)
CPP-D / ODSP	29 (30.9)	22 (31.9)
EI / EI Sick Benefits	6 (6.4)	5 (7.2)
Other (e.g. Ontario Works)	24 (25.5)	17 (24.6)
No response	30 (31.9)	21 (30.4)
Total	94	69
**Housing**		
Owns residence	20 (21.3)	14 (20.3)
Rents residence	58 (61.7)	45 (65.2)
Lives with friends or family	8 (8.5)	5 (7.2)
Other	8 (8.5)	5 (7.2)
Total	94	69
**Relationship Status**		
Married	16 (17.8)	14 (21.5)
Common law or cohabiting	10 (11.1)	4 (6.2)
Single (never married)	31 (34.4)	20 (30.8)
Widowed	2 (2.2)	2 (3.1)
Divorced	17 (18.9)	15 (23.1)
Separated	14 (7.1)	10 (15.4)
Total	90	65
**Citizenship Status**		
Canadian Citizen	77 (88.5)	53 (85.5)
Other	10 (11.5)	9 (14.5)
Total	87	62
**Race**		
White/Caucasian	63 (71.6)	45 (69.2)
Other	25 (28.4)	20 (30.8)
Total	88	65

Notes: ^1^ Participants with legal needs indicated by the Legal Health Check-Up survey; ^2^ *p*-value from Fisher’s Exact Test; *CPP-R* Canadian Pension Plan, retired, *CPP-D* Canadian Pension Plan, disability, *ODSP* Ontario Disability Support Plan, *EI* Employment Insurance

**Table 5:** Poverty indicators, self-reported health status, and quality of life in participants who booked and attended a Legal Health Clinic appointment

	Booked appointment ***n*** = 94	Attended appointment ***n*** = 69
	n (%)	n (%)
**Household Income: Low-Income Measure 50**^**3**^		
Above LIM 50	4 (12.5)	3 (11.1)
Below LIM 50	28 (87.5)	24 (88.9)
Total	32	27
**Income Insecurity**		
Yes	74 (78.7)	54 (78.3)
No	20 (21.3)	15 (21.7)
Total	94	69
**Afford to Buy Medication**		
Yes	38 (40.4)	29 (42.0)
No	56 (59.6)	40 (58.0)
Total	94	69
**Food Insecurity**		
Yes	51 (58.0)	37 (56.9)
No	37 (42.0)	28 (43.1)
Total	88	65
**Housing Insecurity**		
Yes	32 (36.8)	25 (39.1)
No	55 (63.2)	39 (60.9)
Total	88	64
**Self-Reported Health Status**		
Excellent/Very good	11 (12.4)	5 (7.7)
Good/Fair	51 (57.3)	44 (67.7)
Poor	27 (30.3)	16 (24.6)
Total	89	65
**Quality of Life**		
**Mobility**		
No problem	35 (41.2)	22 (34.4)
Some/Severe problems	50 (58.8)	42 (65.6)
Total	85	64
**Self-care**		
No problem	65 (75.6)	48 (75.0)
Some/Severe problems	21 (24.4)	16 (25.0)
Total	86	64
**Performing usual activities**		
No difficulty	23 (26.7)	14 (21.9)
Some/Severe difficulty	63 (73.3)	50 (78.1)
Total	86	64
**Pain and discomfort**		
None	13 (15.1)	9 (14.1)
Some/Severe	73 (84.9)	55 (85.9)
Total	86	64
**Anxiety and depression**		
None	18 (21.2)	16 (25.4)
Some/Severe	67 (78.8)	47 (74.6)
Total	85	63

Note: ^1^ Participants with legal needs indicated by the Legal Health Check-Up survey; ^2^ *p*-value from Fisher’s Exact Test; ^3^Low Income Measure 50 (LIM-50) is the threshold for median (50th percentile) household income, adjusted for household size. Household size was asked on a follow-up survey, not baseline, so LIM-50 could only be calculated for 32 participants

**Table 6:** Types of legal needs identified in participants booked and attended a Legal Health Clinic appointment

Type of Legal Need	Booked appointment ***n*** = 94	Attended appointment ***n*** = 69
	n (%)	n (%)
**Income**	**82 (87.2)**^**a**^	**62 (89.9)**^**a**^
‘Trouble making ends meet’	74 (78.7)	54 (78.3)
Needing help getting or keeping benefits	53 (56.4)	39 (56.5)
Someone taking their money or possessions without permission	21 (22.3)	14 (20.3)
Medical review date for ODSP	5 (5.3)	4 (5.8)
Other/unspecified income issue	37 (39.4)	30 (43.5)
**Housing**	**67 (71.3)**^**a**^	**48 (69.6)**^**a**^
Problems with home repairs, heat not working, bed bugs, etc.	35 (37.2)	25 (36.2)
Denied a rental unit due to discrimination	27 (28.7)	19 (27.5)
Late with rent this year	24 (25.5)	14 (20.3)
Being harassed or discriminated by their landlord	18 (19.1)	14 (20.3)
Worried rent subsidy will be cancelled	13 (13.8)	9 (13.0)
Being threatened with eviction	11 (11.7)	6 (8.7)
Served eviction papers	8 (8.5)	6 (8.7)
Court order affecting where or with whom they can live	8 (8.5)	5 (7.2)
Behind with rent	7 (7.4)	3 (4.3)
Other/unspecified housing issue	23 (24.5)	17 (24.6)
**Employment**	**66 (70.2)**^**a**^	**52 (75.4)**^**a**^
Has been hurt at work	38 (40.4)	30 (43.5)
Having trouble finding work due to discrimination	32 (34.0)	25 (36.2)
Current or past employer owes them money	9 (9.6)	9 (13.0)
Has been harassed by your employer or colleagues	9 (9.6)	8 (11.6)
Other/unspecified employment issue	16 (17.0)	15 (21.7)
**Health**	**53 (56.4)**^**a**^	**41 (59.4)**^**a**^
Does not have someone to make health decisions if they are unable to do so	49 (52.1)	37 (53.6)
Other/unspecified health issue	13 (13.8)	12 (17.4)
**Family/Community**	**91 (96.8)**^**a**^	**66 (95.7)**^**a**^
Does not have a will	80 (85.1)	59 (85.5)
Relationship where someone tries to control them	46 (48.9)	30 (43.5)
Problems with child support or access	24 (25.5)	14 (20.3)
Going through a divorce or separation	18 (19.1)	14 (20.3)
Trouble bringing family members to Canada	13 (13.8)	9 (13.0)
Trouble attaining citizenship	5 (5.3)	5 (7.2)
Other/unspecified family or community issue	14 (14.9)	11 (15.9)

*Note:* ^*a*^ Respondent indicated at least one item from the respective list

In the 69 participants who did attend the LHC, the majority were female (59.4%, *n* = 41), did not complete post-secondary education (59%, *n* = 36), were not employed full-time (89.1%, *n* = 57), were receiving benefits (69.6%, *n* = 48), did not own their residence (79.3%, *n* = 55), were not married or common-law (72.3%, *n* = 47), were Canadian citizens (85.5%, *n* = 53), and were white (69.2%, *n* = 45). The 18–24 year old age category had the lowest representation (7.4%, n = 5) and both the 35–44 and 45–54 year old categories had the highest representation (25.0% and *n* = 17 for both age categories). Please see Table 4 for the full demographic profile.

The poverty indicators were common among LHC attendees (see Table 5), with 78.3% (*n* = 54) reporting income insecurity, 58.0% (*n* = 40) unable to afford their medication, 56.9% (*n* = 37) having food insecurity, and 39.1% (*n* = 25) having housing insecurity. In the subset of 27 respondents who provided household size, 88.9% (*n* = 24) had a household income below the LIM-50 threshold. Self-Reported Health Status in LHC attendees was quite low, with only 7.7% (n = 5) reporting ‘excellent’ or ‘very good’ health, 67.7% (*n* = 44) reporting ‘good’ or ‘fair’ health, and 24.6% (*n* = 16) reporting ‘poor’ health. Quality of life was similarly low among attendees with 85.9% (*n* = 64) reporting pain or discomfort, 78.1% (*n* = 50) difficulty performing their usual activities, 74.6% (*n* = 47) anxiety or depression, 65.6% (*n* = 42) mobility problems, and 25.0% (n = 16) problems with self-care (e.g. dressing unassisted).

Looking at the types of legal needs in attendees (see Table 6), 95.7% (*n* = 66) had a legal need related to family/community, 89.9% (*n* = 62) income, 75.4% (*n* = 52) employment, 69.6% (*n* = 48) housing, and 59.4% (*n* = 41) health. The frequencies for each subcategory of legal need can be seen in Table 6. Notably, 85.5% (*n* = 59) of attendees did not have a will, 78.3% (*n* = 54) had trouble making ends meet, 56.5% (*n* = 39) needed help getting or keeping their benefits, 53.6% (*n* = 37) did not have someone to make health decisions if they become unable, 43.5% (*n* = 30) had been hurt at work, 43.5% (n = 30) were in a relationship where someone was trying to control them, 36.2% (*n* = 25) had problems with their home (e.g. heat not working), and 36.2% (n = 25) were having trouble finding work due to discrimination.

Logistic regression was used to determine the demographic characteristics, poverty indicators, health status, and quality of life domains associated with a participant choosing to attend the LHC or not. Due to a high correlation between employment status and receiving benefits, only employment status was retained in the model. All participants who had a legal need and complete data for the modelled variables were included in the analysis (*n* = 561; see Table 7). Participants with legal needs had significantly higher odds of attending the LHC if they were an ethnicity that was not white (OR = 2.48; 95% CI 1.14–5.39), did not have Canadian citizenship (OR = 4.40; 95% CI 1.48–13.07), had housing insecurity (OR = 3.33; 95% CI 1.53–7.24), and had difficulty performing their usual activities (OR = 2.83; 95% CI 1.08–7.43).

**Table 7:** Logistic regression of participant characteristics associated with attending the Legal Health Clinic

Variable		Attended the Legal Health Clinic (n = 59) Versus Did not attend (*n* = 502)		
		OR	95% CI	***p-value***
**Gender**	Female	REF	–	–
	Male	1.11	0.55, 2.26	0.766
	Transgender	6.74	0.69, 66.30	0.102
**Age Category**	18–24 years	REF	–	–
	25–34 years	0.75	0.19, 3.03	0.686
	35–44 years	1.18	0.31, 4.53	0.811
	45–55 years	0.69	0.18, 2.68	0.592
	55–64 years	1.02	0.24, 4.43	0.976
	65 and older	0.95	0.18, 4.93	0.950
**Ethnicity**	White	REF	–	–
	Other ethnicity	2.48	1.14, 5.39	**0.022**
	No response	6.04	0.338, 96.49	0.204
**Citizenship**	Canadian	REF	–	–
	Other citizenship	4.40	1.48, 13.07	**0.008**
	No response	1.02	0.05, 23.27	0.989
**Relationship Status**	Married/Common Law	REF	–	–
	Other response	1.01	0.45, 2.24	0.988
**Education**	High School or below	REF	–	–
	Any post-secondary	0.99	0.45, 2.18	0.976
	No response	7.97	0.46, 139.21	0.155
**Employment**	Employed	REF	–	–
	Not employed	1.25	0.55, 2.83	0.594
**Monthly Household Income**	Less than $650	REF	–	–
	$700 - $1800	0.62	0.17, 2.18	0.446
	$1850 - $3000	0.36	0.09, 1.51	0.162
	More than $3000	0.36	0.07, 1.89	0.226
**Housing**	Owns their home	REF	–	–
	Does not own their home	1.48	0.60, 3.63	0.397
**Income Insecure**	No	REF	–	–
	Yes	1.95	0.78, 4.93	0.156
**Food Insecure**	No	REF	–	–
	Yes	1.57	0.70, 3.52	0.276
**Housing Insecure**	No	REF	–	–
	Yes	3.33	1.53, 7.24	**0.002**
**Affords Medication**	Yes	REF	–	–
	No	1.15	0.51, 2.61	0.740
**Self-reported Health Status**	Excellent	REF	–	–
	Very Good	0.55	0.05, 5.86	0.622
	Good	1.29	0.14, 12.30	0.826
	Fair	1.25	0.12, 12.86	0.852
	Poor	1.22	0.11, 13.48	0.874
**Mobility**	No problems	REF	–	–
	Some/Severe problems	1.56	0.65, 3.73	0.321
**Self-Care**	No problems	REF	–	–
	Some/Severe problems	0.76	0.30, 1.97	0.575
**Usual Activities**	No problems	REF	–	–
	Some/Severe problems	2.83	1.08, 7.43	**0.034**
**Anxiety/Depression**	No problems	REF	–	–
	Some/Severe problems	0.91	0.39, 2.13	0.826
**Pain/Discomfort**	No problems	REF	–	–
	Some/Severe problems	1.62	0.54, 4.87	0.392

*Note:* Bolding denotes risk factors significant at *p < 0.05*

As a result of the LHC consultations, 58.0% (*n* = 40) were referred to either LAO or HCLC, depending on their income level and area of law needed, 21.74% (*n* = 15) were referred to a private lawyer, and one case was taken on by the LHC lawyer. This does not include cases that were taken on by lawyers at LAO or HCLC post-referral. Also, 47.8% (*n* = 33) of participants were provided education and 29.0% (*n* = 20) of participants were provided resources.

## Discussion

The FHT-based legal clinic appears to be fulfilling a need by providing more equitable access to legal services for vulnerable populations. To date, the USA has been the primary source of research literature on medical-legal partnerships; although there have also been medical-legal partnerships focused on geriatrics, obstetrics and gynecology, oncology and family medicine in the USA, most have been in the area of pediatrics [1]. The current study describes a novel medico-legal partnership in a Canadian family medicine clinic. Lawyers from LAO and HCLC staffed the program successfully for the 6-month study, provided consultations to 69 patients, and have continued to deliver their services to additional FHT patients through the LHC beyond the study time frame. This study found that four out of every five patients in this primary care setting had at least one legal need that could affect their health, therefore the LHC is playing an important role in addressing this gap in access to legal services and may improve health outcomes in the long term.

The LHC was purposefully developed in collaboration with LAO and HCLC to facilitate both immediate feasibility and long-term sustainability. This ensured that organisational attitudes were positive towards the LHC and so funding was made available. At the time of writing, the service has continued for 3 years, with a slight alteration in the participant flow process. Instead of being approached in the waiting room by a research assistant, they are now identified during new patient registration, at which point patients are asked to complete the screening tool, and those identified with unmet legal needs are asked if they would like an LHC appointment. For existing patients, physicians or other health professionals from the FHT can refer patients to the LHC if a new need is identified. In order to make the LHC more sustainable, and because family physicians felt that they were accurately able to assess potential legal needs, the screening tool was dropped for these types of direct referrals, though new patients to the clinic are all screened by clinic staff using the screening tool. Also, there are posters and on-screen advertisements in the waiting areas to raise awareness among patients that the LHC is available and free for them to access.

In the study setting, a FHT in Hamilton, Ontario, 84% of patients screened had unmet legal needs that could benefit from the LHC intervention, with an average of 3.4 legal needs per patient screened. Given that approximately half of patients in the waiting area had a household income below LIM 50 (poverty threshold), the rate of observed legal needs is similar to those found in studies from the USA and Canada suggesting that each low-income household has three or more legal needs per year [3, 4, 29, 30]. The types of legal issues identified through the Legal Health Check-Up in the current study were family/community, income, housing, employment, and health. These are in line with those identified in the Canadian pediatric medical-legal model, where the most common legal issues encountered were: family concerns, immigration/refugee, education, employment, income and housing [2]. Similarly, a report on the civil legal needs of Ontarians has cited that the most common legal problems identified among low and middle income individuals include: family relationship problems, wills and powers of attorney, housing, real estate, and employment (listed from most common to least common) [29]. In contrast, in the United States, the most common legal issues encountered were social security, health insurance coverage, advance directives, food stamps, family concerns, and housing [1]. These legal needs are quite different from those found in the Canadian studies (the current study and the study in pediatrics), [2] highlighting the differences between the USA and Canadian healthcare contexts and the importance of Canadian-based research on this topic to inform future medical-legal partnerships in Canada.

We also found that those with a legal need were more vulnerable compared to those without, with higher rates of housing insecurity, food insecurity, and income insecurity. It has been suggested in Canada that the vulnerability of this population makes them less likely to seek legal assistance, [4] however trusted healthcare professionals can facilitate this introduction, as observed in the current study. When comparing our study participants who attended the legal clinic to those with legal needs who did not attend, there is a clear difference in SES between the two groups. The population that chose to attend had lower income levels, as well as unstable employment and residence. Participants who were not Canadian citizens had significantly higher odds of attending an appointment than Canadians. These results are consistent with the demographic profile of participants in other studies that have implemented medical-legal partnerships and demonstrate the LHC’s success in providing more equitable access [2, 30]. This is expected as vulnerable populations that are negatively impacted by the social determinants of health are more likely to have unmet legal needs and are unable to afford a lawyer [15, 29]. It is these legal needs, that when addressed, can improve the social determinants of health and result in positive health outcomes [7, 13, 14].

One of the limitations of this paper is that the Legal Clinic was only provided in an academic FHT, located in the inner city, urban setting of Hamilton, Ontario. It is possible that in different settings the results would have been different. It is likely though, that the results of this paper are generalizable to other inner cities settings with similar populations.

The McMaster Family Practice Legal Clinic has important implications for the future health of patients, as well as for clinical practice. The Legal Clinic is taking an “upstream” approach to addressing the social determinants of health by ensuring patients receive timely access to the legal services for which they otherwise may have not received help. By helping patients overcome the barrier in accessing legal services and therefore addressing underlying social causes of adverse health outcomes, and though this paper does not measure health outcomes, it is likely that accessing the Legal Clinic will translate into improved health outcomes for these patients [7, 13, 14]. Future research should prospectively follow patients to fully evaluate this impact on health outcomes. Moreover, the implementation of a Legal Clinic will likely benefit clinical practice. Studies that have implemented Legal Clinics have found that clinicians feel as though they are better advocates for their patients by collaborating with a lawyer and knowing where they can refer them [29, 30]. Other family practices may be an ideal setting in which legal services can be provided to vulnerable individuals in a familiar, trusted setting.

## Data Availability

The data are available from the corresponding author upon reasonable request.

## Abbreviations

CPP: Canada Pension Plan
FHT: Family Health Team
HCLC: Hamilton Community Legal Clinic
LAO: Legal Aid Ontario
LHC: Legal Health Clinic
LIM: Low Income Measure
SES: Socioeconomic Status
